# Some Recent Russian Work in Veterinary Science

**Published:** 1900-02

**Authors:** E. V. Wilcox

**Affiliations:** Washington, D.C.


### SOME RECENT RUSSIAN WORK IN VETERINARY SCIENCE.

By E. V. Wilcox, Ph.D.,
WASHINGTON, D.C.

Russian veterinary journals are not very extensively subscribed for in this country, and in the veterinary journals which are published in this country one sees but few references to the current Russian literature. There are two veterinary journals published in Russia which are of especial value, and which contain for the most part records of original investigations along lines which are most studied at the present time. These journals are *Arch. Vet. Nauk*, published in St. Petersburg, and *Uchen Zapiski Kazan. Vet. Inst.* The following is a list of the titles of the more important articles which appeared in these two journals during 1889:

*Archiv. Vet. Nauk, St. Petersburg*, 1899, *vol. xxix.*

The Production of Immunity Against Rinderpest and the Treatment of this Disease, V. E. Vorontzov and others, pp. 1–30, 119–159, 191–227.

Serum-therapy in Chicken-diphtheria, D. V. Devel, pp. 30–49.

Experiments with Serum-therapy in Contagious Pleuropneumonia of Horses, E. N. Usoltzev, pp. 49–62.

The Problem of Fighting the Siberian Plague with the Kazan Vaccine, P. Drozdov, pp. 86–97.

A Study of the Microörganisms of the Alimentary Tract, Y. I. Uspenski, pp. 160–176.

Herpes Tonsurans in Horses, O. S. Kramarev, pp. 176–182.

Some Cases of the Use of Arecolinum Hydrobromicum, M. L. Blumenfeld, pp. 182–187.

Experimental Data on the Problem of the Susceptibilty of the Camel to Rinderpest, M. Tartakovski, pp. 228–254.

Experimental Inoculation of the Camel with Glanders, E. P. Dzhunkovski, pp. 255–264.

Chinosol as a Disinfectant, E. Turkin, pp. 265–281.

Results of the Measurements of Cattle at the Warsaw Agricultural Exhibition, M. V. Zhravski, pp. 281–287.

The Application of X-rays to Veterinary Practice, M. A. Maltzev, pp. 291–298.

Manure and the Natural Agencies which Disinfect It, S. S. Evsyeenko, pp. 298–306.

A New Pathogenic Microbe (cocco-bacterium æruginosum), I. P. Markov, pp. 306–308.

The Pathological-anatomical Changes Produced in the Organs of Mice and Rats by the Rat-destroying Bacilli, G. S. Kulesh, pp. 309–321.

Extent of the Practical Application of the Pasteur Prophylactic Inoculations Against Hog-cholera, O. I. Markevich, pp. 321–331.

The Treatment of Milk-fever with Iodid of Potash, E. Turkin, pp. 331–334.

A Study of Bacterium Coli Commune, A. V. Byelitzer, pp. 339–364.

Treatment of Canine Distemper, M. A . Maltsev, pp. 364–367.

The Influence of Veratrin and Ergot upon Asthmatic Breathing which Accompanies Broken Wind, I. L. Prushkovski, pp. 376–370.

The Influence of the Blood Serum of Horses upon Guinea-pigs, M. N. Zhezhelenko, pp. 423–453, 479–494.

The Diagnosis of Glanders, V. M. Sulin, pp. 454–473.

Anomalous Structure of the Foot in Pigs, N. Dikovski, pp. 473–475, Figs. 2.

Materials for the Study of the Mallein Problem, A. A. Kraevski, pp. 495–516, 519–538.

Death in Horses from Blood Extravasation into the Spinal Canal, L. Doroshenko, pp. 517, 518.

Epizootic Infectious Lameness (hemorrhoids) in Horses, Sotzevich, pp. 381–388.

The Use of Sulphur in the Treatment of Wounds, A. I. Verevkin, pp. 388–395.

Recent Data on the Problem of the Maintenance-ration of Horses, I. Shirokikh, pp. 395–418.

A Case of Carcinoma Hepatis et Renis in a Mare, L. G. Krushinski, pp. 418–420.

A Case of Fracture and Resection of the Rib with Good Result, D. Bliznakov, pp. 420–422.

Parturient Apoplexy in Cows, L. Ivanov, pp. 561–564.

The Illumination of Stables for Domestic Animals and its Influence upon Diseases of the Eye, Tomarov, pp. 552–555.

The Problem of the Influence of the Sex of Horses upon their Susceptibility to Disease, A. Ryazhev, pp. 539–553.

*Uchen Zapiski Kazan. Vet. Inst.*, 1899, *vol. xvi.*

The Pathological-anatomical Changes in the Spinal Cord in Canine Distemper, K. Bol, pp. 1–21, 45–91, 115–228, Plates 2.

Operative Treatment of Scrotal Hernia in Horses, V. A. Glasko, pp. 22–35.

A Simplified Method of Obtaining Corrosion Preparations of the Internal Ear, S. E. Puchkovski and V. V. Zavyatov, pp. 229–243.

Materials for the Study of Hemorrhagic Septicæmia, (swine-plague), K. Z. Kleptzov, pp. 247–334.

Inflammation of the Atlanto-occipital Mucus Capsule, P. Dizderev, pp. 443–451.

This list of titles includes only such articles as represent original and experimental work. Some of them, as, for instance, the article on swine-plague, the article on the changes in the spinal cord during canine distemper, and the article on the mallein problem, are among the most thorough and exhaustive articles on the subjects which are to be found. In a brief note like the present one it is obviously impossible to give the substance of these articles, some of which are of great length. The essential points of such of these articles as are of agricultural importance are given from time to time by the writer in the Experiment Station *Record*. The present list is compiled merely for the purpose of calling attention to this mass of important literature which is much neglected in the United States.

